# A single report tells the story: Ventricular fibrillation caused by vasospastic angina recorded from an implantable loop recorder

**DOI:** 10.1016/j.jccase.2025.05.008

**Published:** 2025-06-11

**Authors:** Daiki Nakajima, Hitoshi Mori, Kazuhisa Matsumoto, Yoshifumi Ikeda, Ritsushi Kato

**Affiliations:** Department of Cardiology, Saitama Medical University, International Medical Center, Saitama, Japan

**Keywords:** Implantable loop recorder, Ventricular fibrillation, Coronary spastic angina

## Abstract

Implantable loop recorders (ILRs) are essential for diagnosing unexplained syncope, particularly when cardiogenic causes are suspected. An 80-year-old male experienced unexplained syncope following chest tightness, with no obstructive coronary findings. A comprehensive cardiac evaluation failed to identify the underlying cause, however Holter electrocardiography showed non-sustained ventricular tachycardia, suggesting cardiogenic syncope. Therefore, we proceeded with ILR implantation. Ten days post-discharge, ILR monitoring detected ventricular fibrillation (VF) preceded by ST-T elevation and triggered by a premature ventricular contraction, confirming vasospastic angina (VSA) as the cause. This is the first documented case where VF due to VSA was diagnosed via a single ILR electrogram. ILRs thus play a crucial role in managing syncope.

**Learning objective:**

Implantable loop recorder (ILR) is useful not only for diagnosing arrhythmias in unexplained syncope, but also for identifying underlying conditions causing the arrhythmias. In this case report, ventricular arrhythmias following ST-T elevation caused by vasospastic angina were detected through a single ILR report.

## Introduction

Implantable loop recorders (ILRs) have been widely used to evaluate the cause of unexplained syncope by continuously recording the heartbeat. Clinical guidelines recommend the use of ILRs as a Class I indication for cases of unexplained syncope suspected to be cardiogenic syncope [1]. By recording the electrocardiogram at the time of the syncope, ILRs enable the analysis of arrhythmias that occur during the event, making it useful for a diagnosis. Several reports have noted the utility of ILRs for diagnosing syncope, and a few reports have revealed the utility of ILRs for ventricular arrhythmias [2,3]. Nakai et al. reported a case of syncope caused by vasospastic angina (VSA), in which an ILR was useful for the diagnosis [4]. In that report, atrioventricular block following ST-T elevation was detected by the ILR, contributing to the diagnosis. However, to our knowledge, there have been no reports about ventricular fibrillation (VF) following ST-T elevation caused by VSA that was diagnosed by a single ILR electrogram.

## Case report

**Fig. 1:**
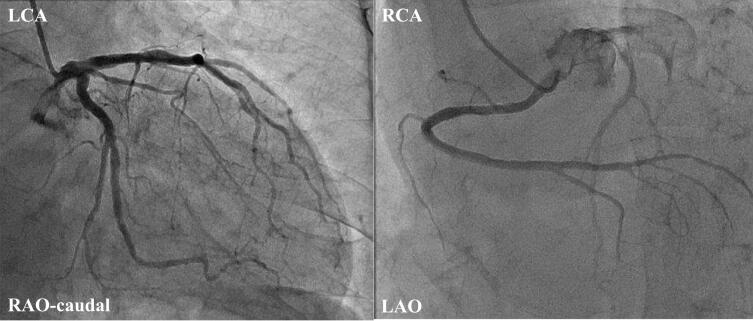
Coronary angiography shows no obstruction of the left coronary artery (left panel) and right coronary artery (right panel). LAO, left anterior oblique; LCA, left coronary artery; RAO, right anterior oblique; RCA, right coronary artery.

**Fig. 2:**
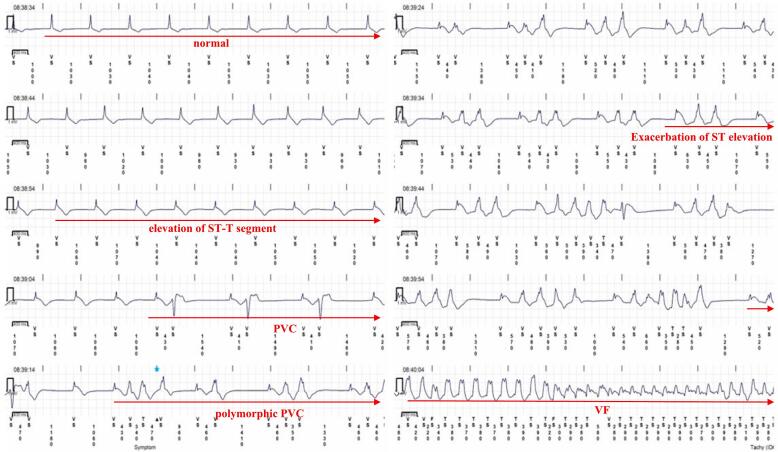
Electrogram recorded by implantable loop recorder. After the initially normal ST-T segment exhibited a mild elevation, multiple PVCs appeared, progressing to polymorphic PVCs. This was followed by an exacerbation of the ST elevation, which finally led to VF. PVC, premature ventricular contraction; VF, ventricular fibrillation.

An 80-year-old male presented to a previous hospital for further evaluation of chest tightness and unexplained syncope. The syncope had recurrently occurred following episodes of chest pain lasting several minutes, raising suspicion of coronary ischemia as the underlying cause. The patient underwent evaluation including transthoracic echocardiography, Holter electrocardiography, and coronary angiography. Transthoracic echocardiography showed a preserved ejection fraction of 65 %, with no wall motion abnormalities or hypertrophy. Holter electrocardiography revealed no ST-segment changes or non-sustained ventricular tachycardia during hospitalization. Coronary angiography was subsequently performed and showed no obstructive lesions (Fig. 1). Furthermore, no ST-segment changes or ventricular arrhythmias were observed throughout the hospital stay. Therefore, cardiogenic syncope due to arrhythmias was suspected, and he was referred to our hospital for further evaluation of the syncope. An ILR was implanted for the evaluation of the unexplained syncope based on the clinical guidelines. Ten days after discharge, VF was reported in the early morning by the alert system of the remote monitoring system. He was urgently hospitalized and the electrocardiogram recorded by the ILR was analyzed, showing that VF was triggered by a premature ventricular contraction, preceded by ST-T elevation (Fig. 2). Given that the event occurred in the early morning, ischemic changes with ST-T elevation were positive, and no significant stenosis was found on coronary angiography, this case was diagnosed as VF caused by variant angina due to VSA. After this diagnosis, calcium channel blockers and isosorbide dinitrate were initiated. Since starting the medical therapy, he has not experienced any syncope or lethal arrhythmia events during the ILR monitoring.

## Discussion

With the miniaturization of ILRs, they play an important role in the management of syncope. ILRs can diagnose the cause of syncope more effectively than conventional syncope evaluations, including electrophysiological testing, in cases of unexplained syncope [5]. Previous reports have also noted that ILRs are useful for detecting syncope caused by VSA and for diagnosing atrioventricular block associated with VSA following ST-T elevation [4]. ILRs are valuable not only for identifying arrhythmias as the cause of syncope but also for diagnosing the underlying condition. However, to our knowledge, this is the first report of VF caused by VSA that was diagnosed through a single ILR electrogram. A single recording from an ILR revealed everything.

## Conclusion

ILRs are invaluable for diagnosing unexplained syncope, providing unique insights into arrhythmias and underlying conditions, as demonstrated in this first reported case of VF caused by VSA.

## Patient consent statement

Patient consent for publication was obtained.

## Consent for publication

Patient consent for publication was obtained.

## Ethics statement

Not applicable.

## Funding information

None.

## Authors' contributions

DN and HM drafted the manuscript; KM and YI revised the manuscript; RK supervised the study.

## Clinical trial registration

Not applicable.

## Declaration of competing interest

HM received lecture fees from Biosense Webster Japan and Boston Scientific Japan. Our department also received grant support from Boston Scientific Japan and Abbott Medical Japan.
